# Mortality in a cohort of transport for London workers

**DOI:** 10.1038/s41598-026-45200-1

**Published:** 2026-03-25

**Authors:** Justie Mak, Johanna Feary, André F. S. Amaral, Emma Marczylo, Paul Cullinan, David C. Green

**Affiliations:** 1 National Heart and Lung Institute, Imperial College London, London, UK; 2 Department of Occupational and Environmental Medicine, Royal Brompton Hospital, London, UK; 3 NIHR Imperial Biomedical Research Centre, Imperial College London, London, UK; 4 Toxicology Department, UK Health Security Agency, Harwell Campus, Chilton, Oxfordshire UK; 5 London Centre for Work and Health, London, UK; 6 MRC Centre for Environment and Health, Environmental Research Group, Imperial College London, London, UK

**Keywords:** Occupational health, Transport, Particulate matter, Diseases, Environmental sciences, Health care, Health occupations, Medical research, Risk factors

## Abstract

Transport workers face various occupational hazards, however long-term effects on mortality are less understood. Transport for London (TfL) employs almost 30,000 workers across a wide range of transport-based jobs and working environments. This study aimed to characterise mortality among TfL employees, and investigate long-term health outcomes. A retrospective cohort was formed using cause of death data from the TfL pension fund for employees working between 1960 and 2010. Workers were grouped by job title and Cox proportional hazard models were used to assess all-cause, respiratory, cardiovascular, and cancer mortality. Bus (hazard ratio HR 1.17, 95% confidence interval CI 1.09–1.25) and London Underground (LU) (HR 1.23, 95% CI 1.15–1.32) workers had significantly higher risks of all-cause, as well as respiratory, cardiovascular, and cancer mortality when compared to office workers. Mortality rates did not differ significantly between bus and LU workers, potentially due to shared occupational or lifestyle risk factors. In this large subway cohort study, mortality rates over 50 years were greater among bus and LU workers compared to office employees. However, findings should be interpreted cautiously due to limitations in data availability and unmeasured confounders. Future prospective studies should address these limitations by collecting detailed health and exposure data.

**Supplementary Information:**

The online version contains supplementary material available at 10.1038/s41598-026-45200-1.

## Introduction

Transport for London (TfL) is the transport authority in London, responsible for providing transportation services across London, including the London Underground, London Overground, buses, trams, river services, cycling, and road networks. In 2023, TfL facilitated approximately 26.1 million journeys every day, 8.6 million of which were made through public transportation^1^. TfL employs over 29,000 employees across a wide range of occupations^2^. Some of the occupations involve specialised training and work environments^3^.

Transport workers are exposed to a range of occupational hazards. This can include environmental exposures, such as noise^4^, and high concentrations of air pollution^5,6^, as well as job-specific factors, such as irregular shift work^7^, customer interactions, and high levels of responsibility^8^. The job can also include physical stressors, such as the sedentary nature of certain jobs, as well as working in confined environments^9^.

Previous studies of transport workers have reported mixed results as to whether they are at a greater risk of mortality compared to the general population. A study of Paris metro workers from 1980 to 2012 found a lower rate of all-cause mortality amongst employees compared to the general population. Furthermore, train drivers, the group with higher occupational exposure to subway particulate matter (PM), had no excess respiratory, cardiovascular, or lung cancer mortality^10^. On the other hand, a study of bus drivers in Genoa, Italy, reported an increased rate of mortality from lung cancer and Hodgkin’s lymphoma, which they attributed to long-term occupational exposure to air pollution^11^. Other research has found that exposure to transportation noise, from railways, aircrafts, and road traffic were associated with increases in cardiovascular, ischemic stroke, myocardial infarction mortalities^12^. Between March and May 2020 during the COVID-19 pandemic, bus and coach drivers in London were at a significantly greater risk of all-cause mortality (128 per 100,000) than all other occupations (78 per 100,000) and double the mortality of bus and coach drivers in the previous five years^13^. The excess risk was likely due to frequent contact with members of the public, underlying morbidities, and workforce demographics. However, the long-term effects on mortality from regular occupational hazards are not known.

Despite the large scale and importance of the TfL workforce, the long-term health outcomes are less well understood, in particular, the pattern of mortality relating to different occupational roles and job categories across TfL. The aim of this study is to characterise and describe mortality patterns in the TfL workforce and assess whether certain occupational groups experience higher rates of mortality compared to others.

## Results

### Cohort characteristics

After excluding job categories that were outside of the study scope and records with missing data, the final cohort size was 117,166.

Table 1 shows that 82% of the cohort were men (95,572), and 43% (50,056) were below 30 years old when they joined. 58% (68,158) were office workers, 13% (15,154) worked in London Buses, 7% (8,143) were engineers, and 22% (25,711) were LU workers. The number of joiners were broadly evenly distributed across the five decades. As of 1 October 2021, 37,849 members (32%) were deceased.

**Table 1:** Characteristics of the study population.

Variable	Frequency of risk factor in study sample	
	*N* = 117,166	%^a^
Sex		
Female	21,594	18.4
Male	95,572	81.6
Age at join		
< 30	50,056	42.7
30–39	27,413	23.4
40–49	22,387	19.1
50–59	13,546	11.6
> 59	3,764	3.2
Job category		
Office	68,158	58.2
Buses	15,154	12.9
Engineers	8,143	7.0
London underground	25,711	21.9
Decade of joining		
1960	22,319	19.0
1970	27,966	23.9
1980	24,357	20.8
1990	27,289	23.3
2000	15,235	13.0
Employment duration (years)^b^		
< 5	41,560	35.5
5–9	20,724	17.7
10–19	33,746	28.8
20–29	15,083	12.9
30–39	4,943	4.2
> 40	1,110	0.9
Vital status^b^		
Living	79,317	67.7
Deceased	37,849	32.3

^a^Prevalence, % in total study sample (*N* = 117,166).

^b^On 1 October 2021.

The distribution of men and women who were deceased were similar to the overall cohort, with 81% (30,824) being men (Table 2). The deceased joined TfL earlier in comparison to the total cohort, with a greater proportion joining in 1960 (47%) and 1970 (39%) compared to the total cohort (19% and 24%). The duration of employment for those in the deceased population was longer than in the overall cohort; 47% of the deceased had worked at TfL for 10–19 years by the time they died compared to 29% in the total cohort.

**Table 2:** Characteristics of the deceased in the study.

Variable	Frequency of risk factor in deceased	
	*N* = 37,849	%^a^
Sex		
Female	7,025	18.6
Male	30,824	81.4
Age at join		
< 30	5,418	14.3
30–39	6,941	18.3
40–49	12,712	33.6
50–59	9,563	25.3
> 59	3,215	8.5
Job category		
Office	12,018	31.8
Buses	12,083	31.9
Engineers	3,790	10.0
London underground	9,958	26.3
Decade of join		
1960	17,906	47.3
1970	14,681	38.8
1980	3,504	9.3
1990	1,328	3.5
2000	430	1.1
Employment duration (years)^b^		
< 5	3,264	8.6
5–9	6,364	16.8
10–19	17,807	47.1
20–29	8,366	22.1
30–39	1,813	4.8
> 40	235	0.6

^a^Prevalence, % in total study sample (*N* = 37,849).

^b^On 01 October 2021.

### Causes of death

From the deceased population (*n* = 37,849), 41% (*n* = 15,605) had missing causes of death. 36 employees were further excluded as typographical errors made it impossible to determine any details relating to cause of death, resulting in 15,641 (41%) employees unsuccessfully grouped into categories of cause of death. 61% (*n* = 7,353) of office workers had no cause of death, making them the job category with the greatest amount of missing data. This was followed by bus workers (47%, *n* = 5,678). LU workers were missing data for 21% (*n* = 2,043) of the population; engineers had the lowest quantity of missing data (15%, *n* = 569). The greatest amount of missing cause of death data stemmed from those who died prior to 1993 (SI Fig. 2); this coincides with the change in data storage method from microfiche to a Structured Query Language database.

The three most common ICD-10 categories were cardiovascular (18% of all mortality), respiratory (16%), and cancer (12%) (SI Table 1). All other known causes of death made up approximately 13% of total mortality. The causes of death were similarly distributed across all job categories (SI Fig. 3).

### Mortality rates

The highest all-cause mortality rate was seen amongst bus workers, with 797 mortalities per 1,000 bus workers (Table 3), and 2,372 mortalities per 100,000 person years (SI Table 2). The lowest all-cause mortality rate was in office workers. Across job categories, bus employees had the highest cause-specific mortality rates.

**Table 3:** Number of mortalities, all-cause, and cause-specific mortality rates per 1,000 in each job category across the entire 1960–2010 TfL cohort and stratified by sex.

Job category	Population (deceased)	Mortalities (*n*)					All-cause mortality rate (per 1,000)	Respiratory mortality rate (per 1,000)	Cardiovascular mortality rate (per 1,000)	Cancer mortality rate (per 1,000)	Lung cancer mortality rate (per 1,000)	Unknown mortality rate (per 1,000)
		Respiratory	Cardiovascular	Cancer	Lung cancer	Unknown						
All cohort												
Office	68,158 (12,018)	1,161	1,545	963	385	7,353	176	17	23	14	4	108
Buses	15,154 (12,083)	1,813	1,971	1,346	513	5,678	797	120	130	89	34	375
Engineers	8,143 (3,790)	905	954	693	236	569	465	111	117	85	29	70
LU	25,711 (9,958)	2,078	2,238	1,784	635	2,043	387	81	87	69	25	79
Female												
Office	14,466 (4,715)	736	937	524	150	1,882	326	51	65	36	10	130
Buses	1,840 (1,093)	208	248	151	56	298	594	113	135	82	30	162
Engineers	925 (175)	34	39	35	9	44	189	37	42	38	10	48
LU	4,363 (1,042)	217	248	199	74	171	239	50	57	46	17	40
Male												
Office	53,692 (7,303)	425	608	439	135	5,471	136	8	11	8	3	102
Buses	13,314 (10,990)	1,605	1,723	1,195	457	5,380	826	121	129	90	34	404
Engineers	7,218 (3,615)	871	915	658	227	525	501	121	127	91	31	73
LU	21,348 (8,916)	1,861	1,989	1,585	561	1,872	418	87	93	74	26	88

Mortality rates for deaths that could not be coded were high relative to respiratory, cardiovascular, and cancer mortality rates, especially for office workers. This reflected the high proportion of missing data for cause of death amongst office workers.

### Mortality associated with job category

**Fig. 1:**
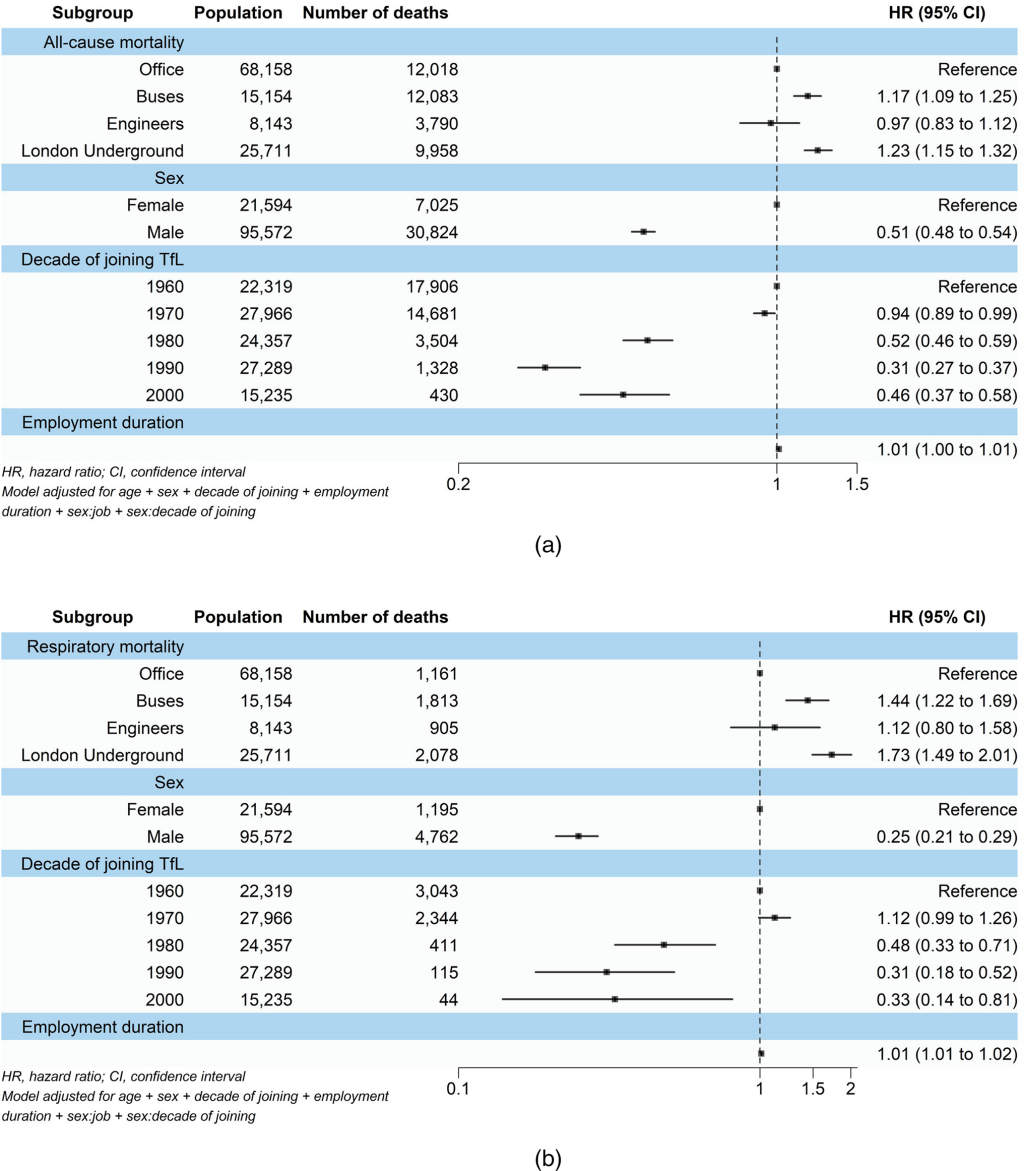

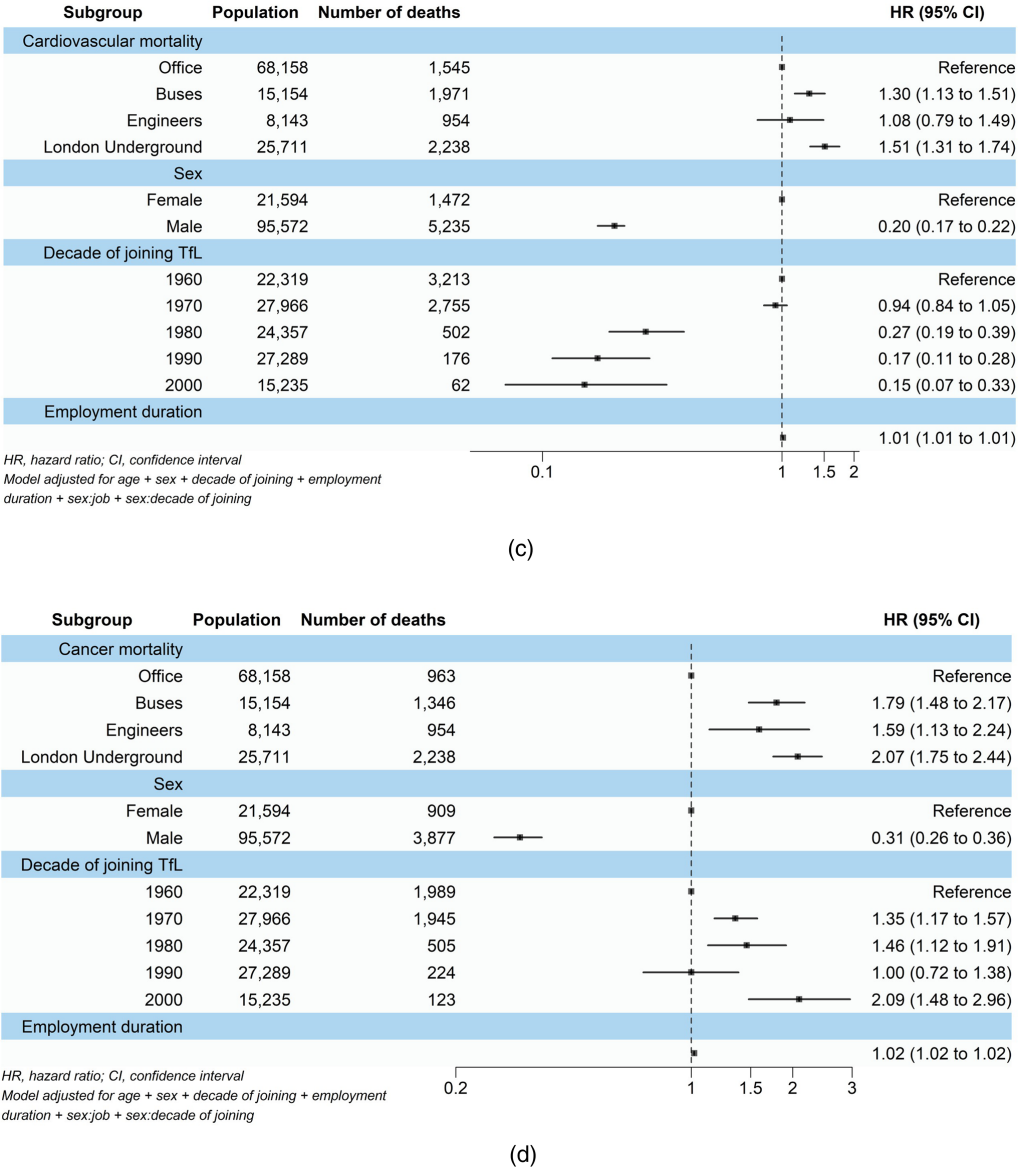

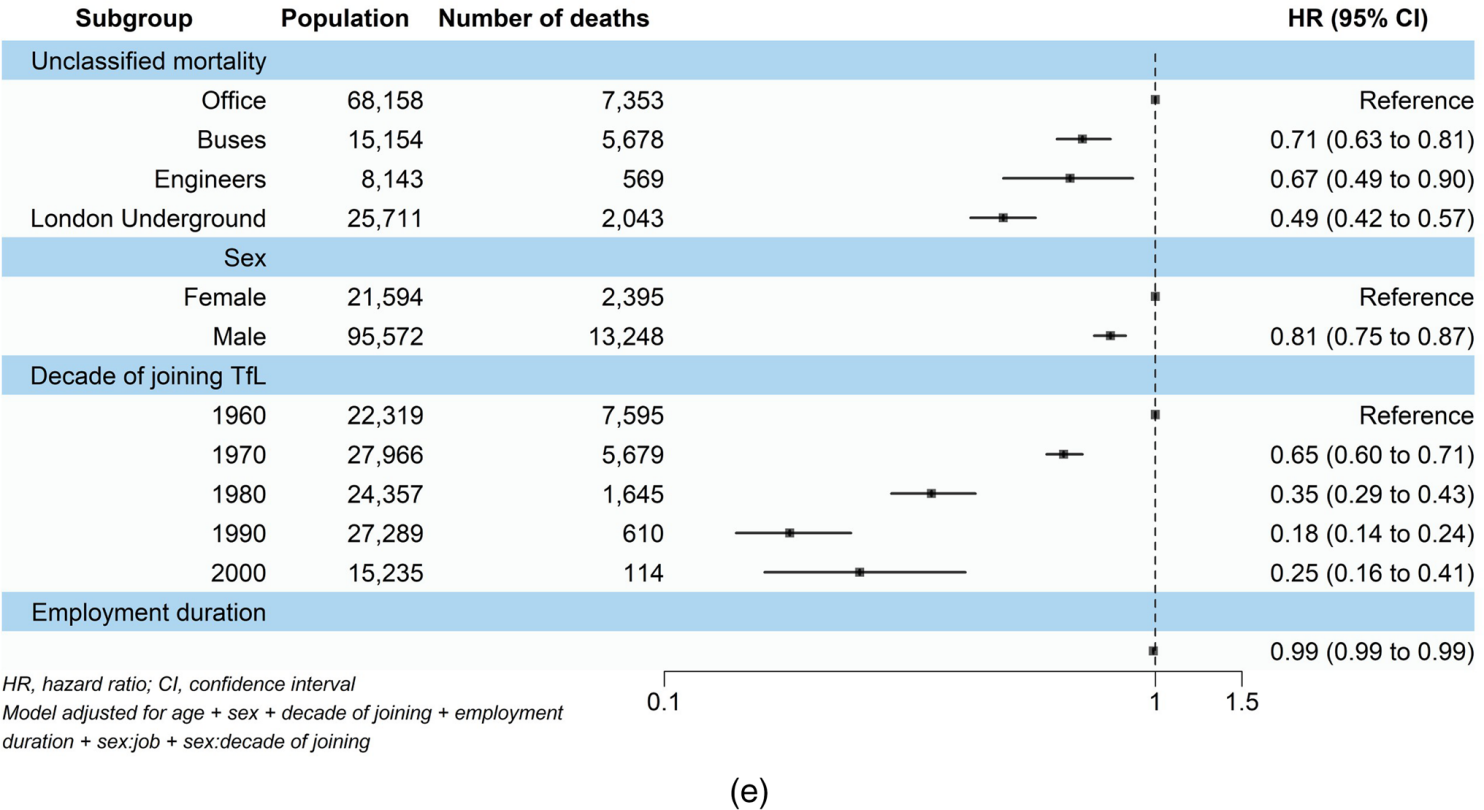
Associations of job category, sex, decade of joining TfL, and employment duration with (**a**) all-cause, (**b**–**d**) cause-specific, and (**e**) unclassified mortality among the 1960–2010 TfL cohort.

Figure 1 shows the results from the main Cox regression model analysis. When looking at all-cause mortality, a 17% (95% CI 1.09, 1.25) increased risk of all-cause mortality was observed amongst bus employees, while LU workers had a 23% (95% CI 1.15, 1.32) higher risk. Engineers showed no significant risk of increased all-cause mortality (HR 0.97, 95% CI 0.83, 1.12). Similar patterns were observed for respiratory and cardiovascular mortality. Bus workers were 44% (95% CI 1.22, 1.69), and LU workers were 73% (95% CI 1.49, 2.02) more likely to experience respiratory-related mortality. Cardiovascular mortality risks were also increased with bus and LU workers showing 30% (95% CI 1.13, 1.51) and 51% (95% CI 1.31, 1.74) increased risks respectively. On the other hand, engineers had no significant increases in respiratory (HR 1.12, 95% CI 0.80, 1.58) and cardiovascular (HR 1.08, 95% CI 0.79, 1.49) mortalities .

**Fig. 2:**
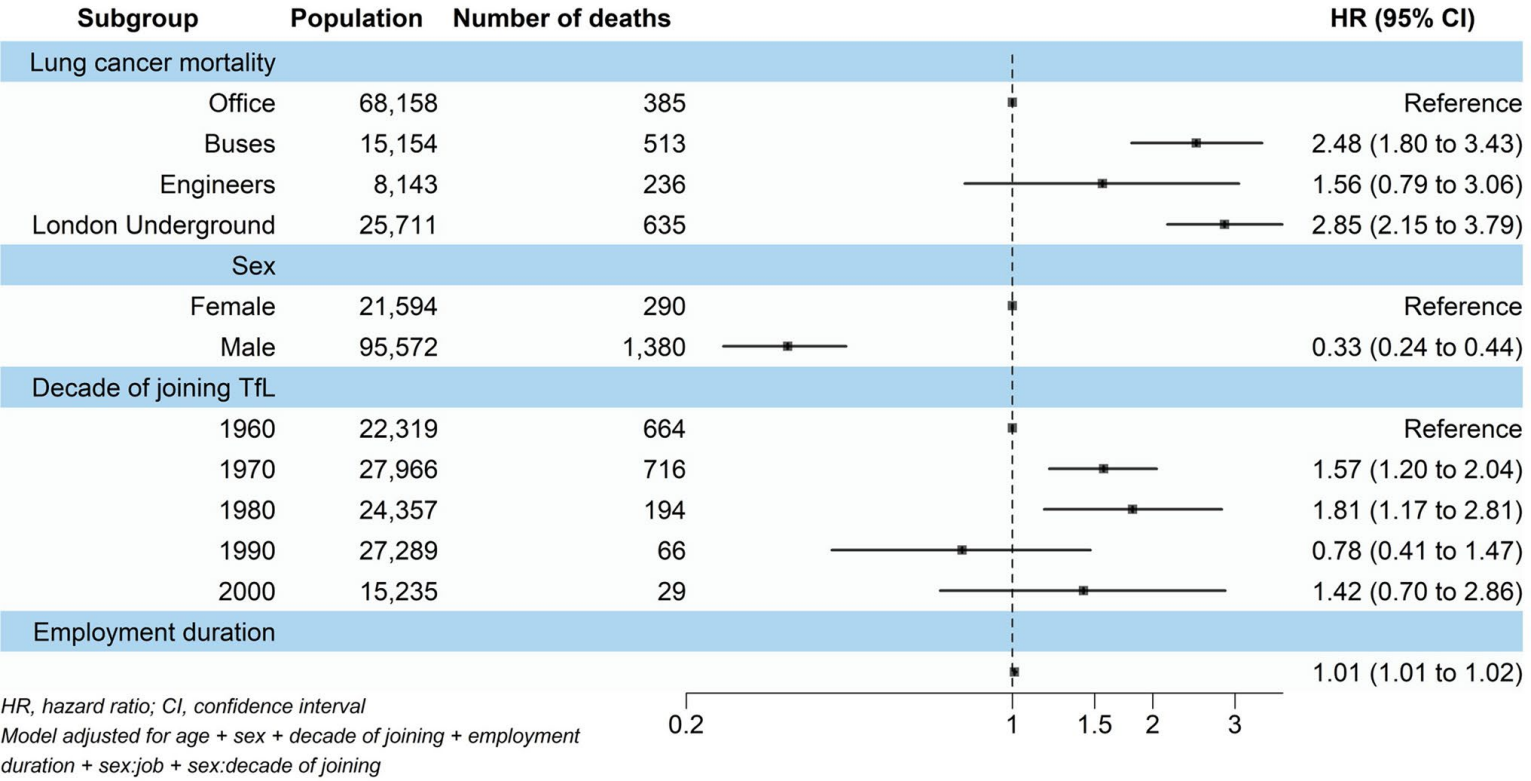
Associations of job category, sex, decade of joining TfL, and employment duration with lung cancer mortality among the 1960–2010 TfL cohort.

All job categories showed significantly higher cancer mortality compared with office workers. Bus workers had a 79% (95% CI 1.48, 2.17) increased risk, engineers a 59% (95% CI 1.13, 2.24) increase, and LU workers more than double the risk (HR 2.07, 95% CI 1.75, 2.44).

On the other hand, bus workers, engineers, and LU workers all showed significantly lower risks of unclassified mortality, with reductions of 29% (HR 0.71, 95% CI 0.63, 0.81), 33% (HR 0.67, 95% CI 0.49, 0.90), and 51% (HR 0.49, 95% CI 0.42, 0.57) respectively.

Across the cohort, there were 1,669 lung cancer mortalities. Bus workers had a 148% (HR 2.48 95% CI 1.80, 3.43) higher risk of lung cancer mortality and LU workers had a 185% (HR 2.85, 95% CI 2.15, 3.79) higher risk compared with office workers. Engineers did not show a significant association (HR 1.56, 95% CI 0.79, 3.06) for lung cancer mortality (Fig. 2).

### Sensitivity analyses

No material differences were seen between the sensitivity analysis excluding all those over 60 years of age and the main analysis (SI Table 3, SI Fig. 4). No meaningful differences to the main findings were seen when excluding any types of cancer apart from lung cancer (SI Fig. 5, SI Table 4, SI Table 5).

## Discussion

### Job-specific mortality trends

This study is the first conducted within a TfL cohort and the largest international subway cohort that investigated patterns of mortality to date. It utilised routinely collected data from the pension fund and had a lengthy follow-up time. 59% of the cohort had complete data to analyse. Bus and LU employees had significantly higher rates of mortality compared to office workers, however findings should be interpreted with caution, due to limitations which are discussed later.

All-cause, respiratory, and cardiovascular mortality risks were higher in bus and LU workers compared with office workers. No increased risk of mortality was observed for engineers.

Bus workers had the highest mortality rate in this study. As the bus service in London was privatised in 1994, no new bus workers joined the cohort after that date. Consequently, older bus workers were over-represented in this cohort, and were therefore more likely to have died compared to the other job categories. However, when limiting analysis of mortality rates to bus and LU workers who joined prior to 1994, there were no significant differences compared to the main analysis.

The higher mortality rates amongst bus employees are consistent with findings from previous occupational health studies. Several cohort studies in New York, Sweden, and Denmark have also reported excess cardiovascular mortality amongst bus drivers. Bus drivers in New York had increased mortality from ischaemic heart disease^14^, and the Swedish and Danish studies found relative risks between 1.5 and 1.6 for myocardial infarction and ischaemic heart disease deaths after adjusting for demographic variables^15,16^. These findings suggests that work-related risk factors, such as stress, exposure to traffic-related air pollution, and sedentary nature of the job may contribute to cardiovascular mortality.

A historical study of transport workers in London found that there was a range of mortality risks depending on their job roles, as has also been demonstrated in this study. Morris, Heady^17^ et al. compared coronary heart disease and mortality in bus drivers and bus conductors working on double decker vehicles. Despite the similar working environments, bus conductors had less coronary heart disease and lower early mortality rates than bus drivers. Coronary heart disease that arose in bus conductors were also less severe than those in bus drivers. This was hypothesised to be due to the sedentary nature of a bus driver’s role compared to the more active role of a bus conductor.

There is less evidence into respiratory mortalities associated with occupational risk factors. A study in Paris found that chronic exposure to subway PM_10_ was associated with an increased risk in COPD, and a decline in lung function being associated with exposure duration^18^. These findings support the results from this study, where an increased risk of cardiovascular, respiratory, and cancer mortality were observed among bus and LU employees is consistent with occupational health risks in the public transport industry.

### Historical mortality

Within this cohort, the most common causes of death were cardiovascular, respiratory, and cancer. From 1990 to 2013, the leading causes of death across England were ischaemic heart disease, cerebrovascular disease, and lung cancer^19^. In contrast, in 2022, leading causes of death were dementia and Alzheimer’s disease^20^. This difference is likely due to different methods used in classifying causes of death. Dementia and Alzheimer’s disease are less likely to be coded in Part 1a of a death certificate. Instead, they would contribute as an underlying disease, presenting in Part 1b, 1c, or 2, therefore going undetected in this study.

The study has several strengths. This is the largest cohort and longest follow up time of all subway studies and is also one of the few studies to report mortality in a subway cohort. The large sample size ensured that there was sufficient power to detect differences in mortality risk between the different job categories. The longer duration of follow-up allowed for greater capture of mortality and a larger dataset. Finally, we carried out an internal comparison of mortality within TfL between both operational and office-based workers.

### Limitations

A key limitation of the study was a lack of granularity in job categories, resulting in substantial heterogeneity across the job categories in both exposure and job titles. Job categories were derived from the Pension Fund database, which records broad job categories rather than detailed or standardised job titles. This limitation was more pronounced for employees who joined the Pension Fund earlier in the follow-up period, as their historical human resources records, which may have contained more detailed job titles, were not available for data linkage. As a result, roles with differing occupational exposure were grouped together and introduced exposure misclassification. Prior evidence has shown that different job grades have varying risks of mortality, with those in lower employment grades being at a greater risk of mortality compared to those in higher grades^21^, suggesting that misclassification of job roles may bias the results towards the null. This limitation was especially relevant for LU employees, as they had a wide range of exposures to LU PM and other hazards depending on their specific jobs. Engineers, while forming the smallest group in the cohort, encompassed the most diverse range of job roles and are likely to have very high levels of heterogeneity. The lack of granular detail about job titles contributes to exposure misclassification and likely will have biased results towards the null. More detailed job titles would have allowed for finer stratification, and the employees would have allowed a more accurate estimation of HRs, as exposure would have been less heterogenous. Additionally, inconsistencies with job title assignments, with first recorded jobs being applied to most cohort members, but job title from the death certificate for those who were deceased, may have further increased exposure misclassification.

Furthermore, the lack of historical quantitative measurements of environmental exposures and other risk factors, including PM, stress, and smoking status, may have resulted in an inability to accurately quantify exposure based on job title, leading to bias in our findings. These constraints reflect the challenges of working with secondary data that were not originally designed for research purposes.

Furthermore, differences in demographics and working practices between the job, such as shiftwork, socioeconomic status, or smoking prevalence, may have influenced mortality. There was a high proportion of missing data on cause of death, particularly for older data. Additionally, the missing data were not evenly distributed across the different job categories. The highest proportion of missing data on cause of death were seen in office workers who formed the reference group in the analysis and may have affected the HR for other job categories for cause-specific mortality. Another limitation was the lack of available data on covariates in the TfL pension dataset which could have confounded the observed associations between exposure and health outcomes. Education, smoking status, alcohol consumption, and stress have been shown to have an effect on mortality^22–26^. Other occupational exposures that an individual may have encountered either before joining or after leaving TfL were also unknown. Furthermore, 72% of the cohort members have an employment duration of less than 20 years, despite the follow-up period of this study being more than 50 years, suggesting that some cohort members may have changed jobs and left TfL, but remained in the Pension Fund. The job, exposure quantity, and duration of exposure may greatly affect an individual’s risk of mortality. For practical reasons we limited data on respiratory, cardiovascular, and cancer mortality to the information from Part 1a of the death certificate only, which limits an understanding of any underlying respiratory, cardiovascular, and cancer diseases that may have been caused by other occupational exposures and may contribute to misclassification in the outcome. Finally, survivor bias may have impacted our results where those who were ill due to effects from occupational health risks may have left their jobs prematurely and be lost to follow up resulting in an underestimation of the association between TfL job history and mortality.

Overall, this study has shown that for the time periods examined bus and LU employees were at greater risk of all-cause mortality, as well as respiratory, cardiovascular, cancer, and lung cancer mortality compared to office workers. However, given a lack of data on important confounders, such as smoking and differences between types of jobs, it is not possible to determine the specific occupational risk factors that contribute to the increased mortality in bus and LU employees. Further research, such as prospective panel and cohort studies, is needed to identify the causal pathways for mortality differences amongst TfL workers.

## Methods

### Study population

A pseudonymised dataset containing employee data for those who joined the TfL Pension Fund between 1960 and 2010 was constructed, and data were extracted for each pension fund member. Cohort follow-up ran from 1 January 1960 to 1 October 2021. Those who joined the TfL Pension Fund prior to 1 January 1960 were not included in the study cohort.

Upon starting employment at TfL, all staff were automatically enrolled into the TfL Pension Fund and remained members even if they changed roles within the organisation or left TfL. Members only exited the fund if they explicitly requested withdrawal or at the time of death. Job-related information recorded in the pension fund primarily reflects the organisation or operational group (e.g., bus, London Underground) at the time of joining. Historical records do not consistently include detailed job titles due to legacy data limitations and missing information. For more recent joiners, detailed job titles were available through the human resources database. For these individuals, pension records were linked to human resources records to derive an exact job title. However, this linkage was only possible for employees who joined from 2007 onwards, as no human resources job-title information exists for earlier joiners. This affects only a small proportion of the cohort, since the 2007–2010 period represents the final three years of entry into the cohort. For deceased members, the job title recorded on the death certificate may reflect the last occupation reported by relatives, which may or may not correspond to their final role at TfL.

Employees were grouped into job categories by expert users at TfL who understood historical jobs and their descriptions^3^. This process involved input from members of the TfL Pension Fund, human resources personnel, occupational health practitioners and hygienists, safety, health, and environment team, and other long-serving employees who were familiar with historical changes in job structures and organisational systems. For those who were deceased, pension records contained copies of their death certificates. Information on date and cause of death was extracted from death certificates.

The initial cohort consisted of 173,237 employees. Employees in the office, engineers, bus, and London Underground (LU) job categories were included in the study. Ferry workers (*n* = 138) and builders (*n* = 635) were excluded, since they were the smallest groups and lacked sufficient power for analysis. Those with missing data for date of birth, date of joining TfL or the pension fund, and job categories were also excluded (SI Fig. 1).

These data were provided by TfL and the TfL Pension Fund for the purpose of this study.

### Job categories

Four job categories were derived based on the jobs that were noted on the death certificates and TfL Pension Fund databases.

Office workers undertook non-operational roles at office buildings separate to the LU network. They were used as a reference group as they were least likely to be exposed to some occupational hazards that other employees would have been exposed to, such as shiftwork, loud noises, and elevated PM concentrations from train wear sources in the LU^27^.

Engineers carried out a wide range of roles, each with its own range of occupational hazards. This group included some engineers, such as civil engineers, unexposed to LU PM and others such as carpenters and welders, who were exposed to other (non-LU) sources of PM. As job titles were not stored within the pension fund database and were only available on death certificates, it was not possible to further distinguish the type of engineer.

Bus workers worked on surface transport and included bus drivers, ticket sellers, and bus conductors. Bus workers share some commonalities and occupational hazards with LU workers, but would have been exposed to other important occupational hazards, such as outdoor PM.

Finally, those in the “LU” job category consisted of everyone who worked in the LU organisation, as exact jobs could not be specified. This included drivers and Customer Service (CS) employees who were routinely exposed to LU PM, office workers who worked in office buildings and were unexposed to LU PM, and engineers, who may have had occupationally exposure to other sources of PM.

### Causes of death

Causes of death were manually transcribed from the death certificates and initially coded by Iris V5 software^28^. A data dictionary was obtained from the Office of National Statistics (ONS), which translated text entries to World Health Organisation International Classification of Disease 10th Revision (ICD-10) codes and ensured consistency in coding. The cause of death was categorised into the type of disease or health condition using the first alphabetical character of the ICD-10 code.

Manual cleaning of Part 1a causes that were not successfully coded by Iris was conducted by a physician. During the manual cleaning, the diseases not coded by Iris were individually grouped into the categories of diseases, as dictated by the first alphabetical character of the ICD-10 codes.

Respiratory and cardiovascular diseases, and cancer were of most interest as they were some of the leading causes of death between 1990 and 2013^19^ and also formed the largest proportion of deaths in this cohort. Underlying cause of death was identified from Part 1a of the death certificates.

Lung cancer was also selected as a disease of interest (C34 in ICD-10) and cases were identified from all parts of the death certificate (parts 1a, 1b, 1c and part 2).

### Statistical analysis

Cumulative mortality rate was calculated for all-cause, respiratory, cardiovascular, and cancer mortality. Cox proportional hazard models were used to investigate the relationship between job category and mortality. The models used age as a time scale, starting at the age of joining the pension fund to account for left truncation of employees in the cohort^6,29^. A separate model was constructed for all-cause and cause-specific mortality as extracted from Part 1a of the death certificates. Another model was constructed with lung cancer mortality as an outcome, as extracted from Parts 1a, 1b, 1c, and 2. In these models, censoring occurred on the date of death, or at the end of the follow-up period (01/10/2021). A final model was constructed for those who were deceased but had unknown causes of death.

Covariates that were included in the models were based on the availability of data. They included sex, decade of joining TfL, and employment duration. Interaction terms were also included for sex and job, and sex and decade of joining.

Mortality based on person-years was also calculated for each job group (SI Table 2).

### Sensitivity analysis

Sensitivity analysis was conducted by limiting the cohort to those aged under 60, including those who were still alive and those who died under the age of 60 to represent premature mortality. The final type of sensitivity analysis examined lung cancer specifically. In the main analysis, any incident of lung cancer was identified and included in the models. In the sensitivity analysis, only those who had lung cancer and no other types of cancer on their death certificate were included. This excluded any cases where lung cancer was caused by metastasis of a different form of cancer.

All statistical analyses were carried out with R software (version 4.2.2).

## Supplementary Information

Below is the link to the electronic supplementary material.

Supplementary Material 1

## Data Availability

The data that support the findings of this study are available from Transport for London, but restrictions apply to the availability of these data, which were used under license for the current study, and so are not publicly available. Data are however available from the authors upon reasonable request and with permission of Transport for London.
