# Supplementary material for: Mortality in a cohort of transport for London workers
Source: Sci Rep. 2026 Mar 25;16:14917. doi: 10.1038/s41598-026-45200-1 (PMC13168242; doi:10.1038/s41598-026-45200-1)
Supplement: Supplementary file 1 — Supplementary Material 1 [file 41598_2026_45200_MOESM1_ESM.docx]

SI Figure 1. Flow chart of cohort exclusion process

Total joined TfL Pension Fund from 1960-2010 (n=173,237)

Cohort after removing job categories out of the study scope (n=172,492)

Job roles out of study scope excluded

Ferry (n=138)

LRT Builders (n-607)

Cohort after removing those with unknown jobs (n=157,360)

Unknown job (missing from Pension Fund database, could not be identified through HR records or death certificate) (n=15,132)

Total cohort included in data analysis (n=117,166)

Missing date of joining the Pension Fund or TfL and missing date of birth (n=40,194)

SI Figure 2. Distribution of missing cause of death data by year of death.

*
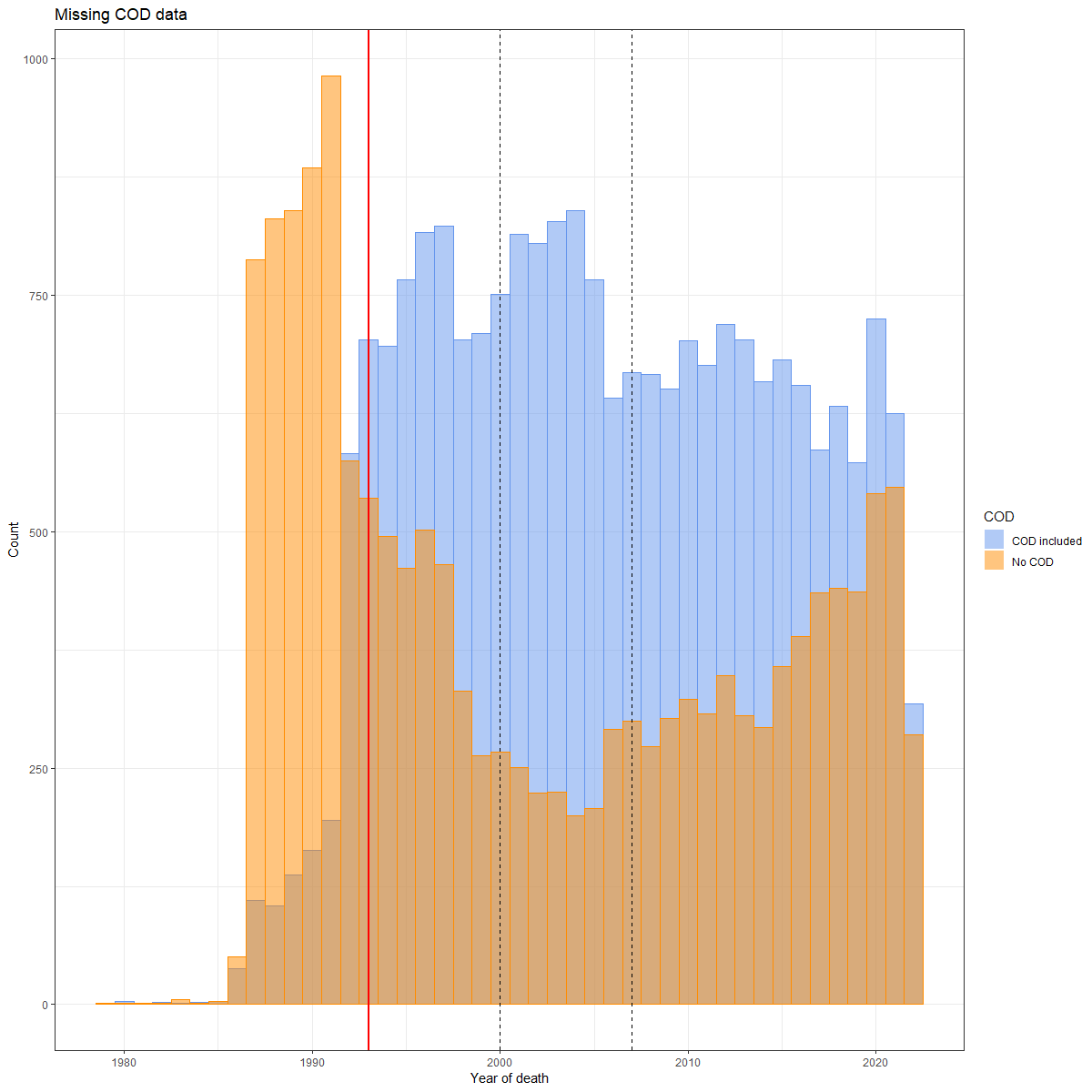
*

SI Table 1. Number and proportion of mortalities with causes of death categorised according to the WHO ICD-10, coded by the Iris software and manually.

| **WHO ICD-10 category** | **Coded by Iris, N (%)** | **Coded by Iris and manually, N (%)** |
| --- | --- | --- |
| Certain infectious and parasitic diseases | 635 (1.7) | 702 (1.9) |
| Neoplasms | 2,182 (5.7) | 4,786 (12.6) |
| Diseases of the blood and blood-forming organs and certain disorders involving the immune mechanism | 19 (0.1) | 37 (0.1) |
| Endocrine, nutritional and metabolic diseases | 56 (0.1) | 68 (0.2) |
| Mental and behavioural disorders | 315 (0.8) | 361 (1.0) |
| Diseases of the nervous system | 262 (0.6) | 441 (1.2) |
| Diseases of the circulatory system | 5,992 (15.8) | 6,708 (17.7) |
| Diseases of the respiratory system | 5,435 (14.3) | 5,957 (15.7) |
| Diseases of the digestive system | 516 (1.3) | 756 (2.0) |
| Diseases of the skin and subcutaneous tissue | 7 (0.0) | 7 (0.0) |
| Diseases of the musculoskeletal system and connective tissue | 6 (0.0) | 8 (0.0) |
| Diseases of the genitourinary system | 440 (1.2) | 491 (1.3) |
| Pregnancy, childbirth and the puerperium | 0 (0.0) | 1 (0.0) |
| Congenital malformations, deformations and chromosomal abnormalities | 1 (0.0) | 1 (0.0) |
| Symptoms, signs and abnormal clinical and laboratory findings, not elsewhere classified | 1,160 (3.1) | 1,410 (3.7) |
| Injury, poisoning and certain other consequences of external causes | 85 (0.2) | 85 (0.2) |
| External causes of morbidity and mortality | 0 (0.0) | 188 (0.5) |
| Codes for special purposes | 149 (0.4) | 201 (0.5) |
| NA | 20,589 (54.7) | 15,641 (41.3) |

SI Figure 3. Distribution of causes of death across the job categories in the 1960-2010 TfL cohort as coded in ICD-10, with NA being those who had missing causes of death.


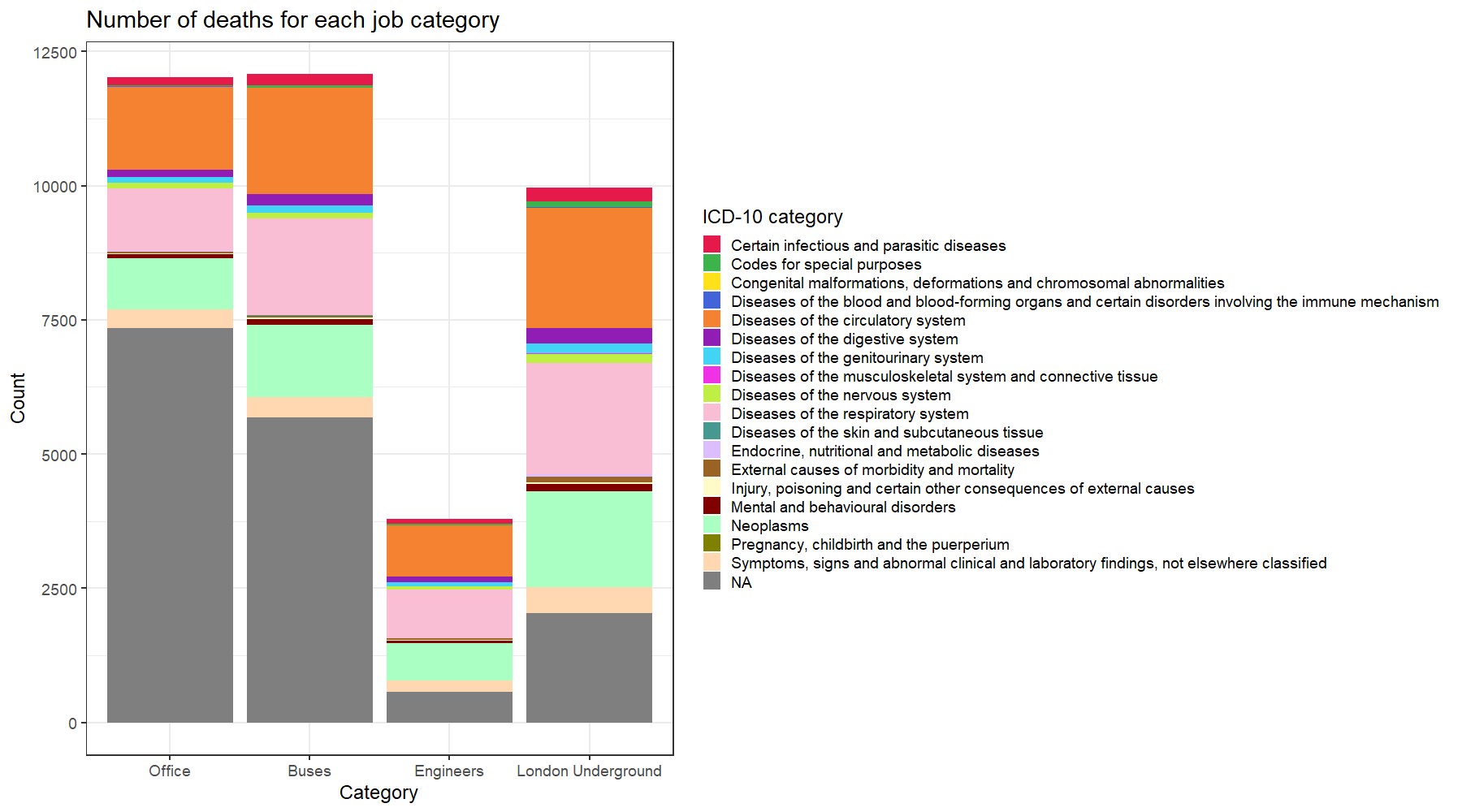


SI Table 2. Mortalities per 100,000 person years

| Job category | Cumulative deaths (n) | Total person years | Mortalities per 100,000 person years |
| --- | --- | --- | --- |
| Office | 12,018 | 2,364,588 | 508.25 |
| Buses | 12,083 | 509,338 | 2372.30 |
| Engineers | 3,790 | 241,676 | 1568.22 |
| London Underground | 9,958 | 804,429 | 1237.90 |

SI Table 3. Number of mortalities, all-cause, and cause-specific mortality rates per 1,000 in each job category across the 1960-2010 TfL cohort under the age of 60 and stratified by sex.

| Job | Population (deceased) | Mortalities (n) | | | | | All-cause mortality rate (per 1,000) | Respiratory mortality rate (per 1,000) | Cardiovascular mortality rate (per 1,000) | Cancer mortality rate (per 1,000) | Lung cancer mortality rate (per 1,000) | Unknown mortality rate (per 1,000) |
| --- | --- | --- | --- | --- | --- | --- | --- | --- | --- | --- | --- | --- |
|  |  | Respiratory | Cardiovascular | Cancer | Lung cancer | Unknown |  |  |  |  |  |  |
| **All cohort** | | | | | | | | | | | |  |
| Office | 57,517 (1,377) | 64 | 171 | 149 | 19 | 875 | 24 | 1 | 3 | 3 | 1 | 15 |
| Buses | 4,227 (1,156) | 95 | 229 | 180 | 44 | 1,156 | 273 | 22 | 54 | 43 | 10 | 123 |
| Engineers | 4,711 (358) | 34 | 110 | 90 | 15 | 358 | 76 | 7 | 23 | 19 | 3 | 10 |
| LU | 17,007 (1,254) | 144 | 306 | 271 | 74 | 1,254 | 74 | 8 | 18 | 16 | 4 | 12 |
| **Female** | | | | | | | | | | | |  |
| Office | 10,033 (282) | 15 | 31 | 57 | 10 | 146 | 28 | 2 | 3 | 6 | 1 | 15 |
| Buses | 827 (80) | 3 | 14 | 16 | 1 | 36 | 97 | 4 | 17 | 19 | 1 | 44 |
| Engineers | 765 (15) | 0 | 1 | 10 | 1 | 2 | 20 | 0 | 1 | 13 | 1 | 3 |
| LU | 3,430 (109) | 6 | 16 | 39 | 6 | 20 | 32 | 2 | 5 | 11 | 2 | 6 |
| **Male** | | | | | | | | | | | |  |
| Office | 47,484 (1,095) | 49 | 140 | 92 | 9 | 729 | 23 | 1 | 3 | 2 | 1 | 15 |
| Buses | 3,400 (1,076) | 92 | 215 | 164 | 43 | 485 | 316 | 27 | 63 | 48 | 13 | 143 |
| Engineers | 3,946 (343) | 34 | 109 | 80 | 14 | 46 | 87 | 9 | 28 | 20 | 4 | 12 |
| LU | 13,577 (1,145) | 138 | 290 | 232 | 68 | 191 | 84 | 10 | 21 | 17 | 5 | 14 |

SI Figure 4. Associations of job category, sex, decade of joining TfL, and employment duration with (a) all-cause, (b-d) cause-specific, and (e) unclassified mortality under the age of 60 among the 1960-2010 TfL cohort.

(a)





0.01

(b)





0.01


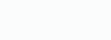


(c)





0.01


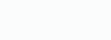


(d)

(d)





0.2


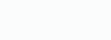


(e)





0.01



SI Figure 5. Associations of job category, sex, decade of joining TfL, and employment duration with lung cancer mortality among the 1960-2010 TfL cohort.

SI Table 4. Number of deaths, all lung cancer, and lung cancer without metastasis mortality rates per 1,000 in each job category across the 1960-2010 TfL cohort and stratified by sex.

| Job category | Population (dead) | Deaths (n) | | Lung cancer mortality rate (per 1,000) | Lung cancer mortality rate, no metastasis (per 1,000) |
| --- | --- | --- | --- | --- | --- |
|  |  | Lung cancer | Lung cancer (no metastasis) |  |  |
| **All cohort** | | | | | |
| Office | 68,158 (12,018) | 285 | 203 | 4 | 3 |
| Buses | 15,154 (12,083) | 513 | 415 | 34 | 27 |
| Engineers | 8,143 (3,790) | 236 | 189 | 29 | 23 |
| LU | 25,711 (9,958) | 635 | 516 | 25 | 20 |
| **Female** | | | | | |
| Office | 14,466 (4,715) | 150 | 101 | 10 | 7 |
| Buses | 1,840 (1,093) | 56 | 50 | 30 | 27 |
| Engineers | 925 (175) | 9 | 5 | 10 | 5 |
| LU | 4,363 (1,042) | 74 | 58 | 17 | 13 |
| **Male** | | | | | |
| Office | 53,692 (7,303) | 135 | 102 | 3 | 2 |
| Buses | 13,314 (10,990) | 457 | 365 | 34 | 27 |
| Engineers | 7,218 (3,615) | 227 | 184 | 31 | 25 |
| LU | 21,348 (8,916) | 561 | 458 | 26 | 21 |

SI Table 5. Associations of job category, decade of joining TfL, and employment duration with mortality from lung cancer without metastasis among the 1960-2010 TfL.

| Variable | HR (95% CI) |
| --- | --- |
|  | Lung cancer deaths with no metastasis |
| Job category | |
| Office | Reference |
| Buses | 3.37 (2.36, 4.81) |
| Engineers | 1.25 (0.51, 3.08) |
| London Underground | 3.35 (2.41, 4.66) |
| Sex | |
| Female | Reference |
| Male | 0.39 (0.27, 0.57) |
| Decade of joining TfL | |
| 1960 | Reference |
| 1970 | 1.67 (1.23, 2.28) |
| 1980 | 2.01 (1.20, 3.37) |
| 1990 | 0.90 (0.43, 1.90) |
| 2000 | 1.75 (0.79, 3.88) |
| Employment duration | 1.02 (1.01, 1.02) |
